# An Explainable Plane-Wise ConvNet Approach for Detecting Femoral Head Osteonecrosis from Magnetic Resonance Images

**DOI:** 10.3390/bioengineering13050529

**Published:** 2026-04-30

**Authors:** Şükrü Demir, Mehmet Vural, Buğra Can, Fatih Demir, Abdulkadir Sengur

**Affiliations:** 1 Faculty of Medicine, Firat University, 23119 Elazig, Turkey; sukru.demir@firat.edu.tr (Ş.D.); b.can@firat.edu.tr (B.C.); 2 Digital Transformation Office, Firat University, 23119 Elazig, Turkey; mvural@firat.edu.tr; 3 INCISOFT Software Information Technologies, Fırat Teknokent, Fırat University, 23000 Elazig, Turkey; fatihdemir@firat.edu.tr; 4 Department of Electrical-Electronic Engineering, Faculty of Technology, Firat University, 23000 Elazig, Turkey

**Keywords:** osteonecrosis of the femoral head, deep learning, Ficat staging, class imbalances, explainable artificial intelligence

## Abstract

**Background/Objectives**: Osteonecrosis of the femoral head (ONFH) is difficult to diagnose, particularly in the early stages, because radiological findings may be subtle. Delayed or inaccurate staging may increase the risk of femoral head collapse and functional loss. Although magnetic resonance imaging is highly sensitive for early-stage lesion detection, interpretation may vary depending on observer experience. Therefore, reliable and explainable automated decision support approaches are needed. **Methods**: In this study, a deep learning-based approach was proposed to classify ONFH into early and late stages according to the Ficat–Arlet staging system. Stage I–II cases were defined as early-stage, whereas Stage III–IV cases were defined as late-stage. Axial and coronal MR images were evaluated separately to investigate plane-dependent classification performance. The images were converted into a three-channel format, resized to a common spatial resolution, normalized, and augmented during training. Feature extraction was performed using transfer learning with modern convolutional neural network architectures. ConvNeXt Tiny was used as the main classification backbone. Weighted loss was applied to reduce the effect of class imbalance, and the decision threshold was optimized on validation data to reduce missed clinically critical late-stage cases. **Results**: A dataset collected from the Orthopedics and Traumatology Department of Firat University Hospital was used in the experimental evaluation. The dataset was divided into training and test sets using an 80:20 split, and 10-fold cross-validation was additionally performed to assess model stability. In the hold-out test, the axial plane model achieved 94.51% accuracy, 96.80% sensitivity, 93.49% specificity, 0.9162 F1-score, and 0.981 AUC. In the coronal plane model, 92.84% accuracy, 96.13% sensitivity, 90.96% specificity, 0.9072 F1-score, and 0.988 AUC were obtained. The 10-fold cross-validation results provided a more conservative estimate of generalization performance. **Conclusions**: The findings indicate that deep learning-based plane-wise analysis of MR images can distinguish early- and late-stage ONFH with high performance. Grad-CAM-based visual explanations showed that the model focused mainly on clinically relevant subchondral and weight-bearing regions of the femoral head. The proposed approach may serve as an explainable decision support tool for reducing observer-dependent variability in clinical staging. Future studies should validate the method using external, multicenter datasets and paired patient-level axial–coronal images.

## 1. Introduction

Femoral head osteonecrosis (ONFH) is a progressive disease, commonly described as avascular necrosis, resulting from decreased and impaired blood flow to the subchondral bone [1]. This ischemic process leads to osteocyte necrosis, microstructural weakening, and often loss of integrity of the spherical structure of the femoral head. This clinical course is particularly important in young and middle-aged adults because femoral head collapse leads to secondary osteoarthritis and usually results in total hip arthroplasty [2]. In cases of late diagnosis, the effectiveness of joint-preserving treatment is significantly reduced, making the process leading to arthroplasty unavoidable [2,3].

The underlying causes of ONFH are heterogeneous and multidimensional. In non-traumatic cases, high-dose corticosteroid uses or prolonged excessive alcohol consumption are the most consistent risk factors. However, metabolic, autoimmune, and hemoglobinopathies, as well as prothrombotic clinical conditions, are also associated with the disease [1,4]. The pathogenesis is multifactorial and involves vascular occlusion, increased intraosseous pressure, bone marrow fat-cell hypertrophy, endothelial injury, and impaired bone remodeling, ultimately leading to subchondral ischemia [4,5]. ONFH should be considered in the differential diagnosis of patients with function-related hip pain, particularly when relevant risk factors are present, even if radiographic findings are inconclusive [2,5].

At this stage, imaging plays a fundamental role in both diagnosis and staging of the disease. Although plain radiographs can provide diagnostically meaningful information after structural deformations have occurred, they may often appear normal in early-stage ONFH cases [2,3]. MRI is adopted as the most sensitive diagnostic method for ONFH, especially in the radiographically inconsistent stage. The ability to reveal the distribution of damaged tissue, subchondral fracture, and early collapse is vital in determining the natural clinical course of the disease [3,5,6,7]. The serpiginous lesion margin and the “double-line sign” on T2-weighted images are well-known diagnostic features [7]. In addition, the most decisive factor in the treatment decision-making process is whether the femoral head collapse has progressed. While femoral head-preserving treatment strategies can be evaluated in the pre-collapse stage, treatment methods after collapse increasingly focus on arthroplasty [2,3,8]. For this reason, the early-late distinction is not only descriptive but also the main determinant of the treatment strategy.

Different staging systems have been proposed for the systematic analysis of ONFH. The Ficat–Arlet classification is widely preferred in clinical practice, and MRI findings are heavily included in this system to contribute to the detection of early-stage disease in current practice [6,8]. The ARCO staging system, revised with international consensus, offers a more structured, image-based framework and is gaining increasing acceptance [8]. However, a significant limitation of staging systems is observer dependence. Previous studies have shown only poor-to-fair reproducibility for both Ficat and ARCO staging systems, with reported inter-observer kappa values of approximately 0.31–0.39 and intra-observer kappa values of approximately 0.43–0.52, particularly in borderline or intermediate cases. This variability makes clinical standardization difficult, reduces reproducibility in research, and further strengthens the rationale for automated decision support systems [9].

Recently, deep learning approaches have played a major role in the classification of medical images [10,11]. Research on musculoskeletal imaging, in particular, is noteworthy [10,11]. In studies using multicenter datasets in the field of ONFH, models capable of performing automated staging and disease severity assessment based on magnetic resonance imaging have been reported to exhibit performance close to expert assessments [12]. Although methods for lesion detection and classification based on CNN for early-stage ONFH have been defined, it is seen that most of these approaches in the current literature do not include a standard staging process [11]. Furthermore, it is observed that holistic models that address early and late stages in a way that directly reflects the classical Ficat stages in our daily practice, interpret many image planes together, and are compatible with clinical decision support systems are limited in the literature [2,3,8,9,11,12].

The main objective of this study is to develop a system that can automatically classify ONFH cases into Ficat stages based on MR slices and make a clinically significant distinction between early (I–II) and late (III–IV) stages [2,5,6,8]. The separate evaluation of axial and coronal sections was performed to investigate plane-dependent classification performance, since lesion visibility and subchondral changes may vary across anatomical planes. The spread characteristics of lesions located in the anterosuperior weighted region and the detection of subchondral depression can show varying characteristics in different image sections [3,8]. The main goal of this approach is to reduce observer-induced variability in ONFH staging and to establish a more repeatable decision mechanism [9,11].

Early and accurate staging of ONFH lesions is crucial for timely access to joint-preserving treatment options. Clinically, the determining threshold is often the difference between the pre-collapse and post-collapse phases. Therefore, reliable detection of early-stage disease is considered a fundamental requirement that shapes not only diagnosis but also treatment. In this regard, numerous studies based on deep learning and artificial intelligence, aiming to overcome the limitations of traditional radiological evaluations, have been published in the literature in recent years. The studies reported in the literature do not exhibit a homogeneous structure in terms of imaging modality, methodological approach, and the type of clinical problem targeted.

Table 1 presents a comparison of deep learning-based studies in the diagnosis of ONFH. The table summarizes the model and methods used in each study, the imaging modalities, performance percentages, and associated limitations. Developments in the literature show that MR imaging methods are far more dominant compared to other methods. The main reason for this is that MRI technology offers high sensitivity in early-stage cases, allowing for the visualization of a critical process such as subchondral insufficiency. One concrete example of this field is the study by Shen et al. [12], which was conducted with a CNN model inspired by the DenseNet architecture for a multi-class severity analysis based on the JIC system. The model, presented with multicenter cross-sectional data, reportedly achieved an accuracy of 87.8% in the expansion internal test sections. However, the retrospective nature of the study and the insufficient representation of the early-stage sample limit its reliability regarding early diagnosis. A similar situation was observed in the study reported by Klontzas et al. [13]; although high AUC and success rates were obtained with the CNN-ensemble approach from STIR sequences, the limited sample size caused a significant decrease in external test performance and raised doubts about the generalizability of the model. In the MRI-based literature, more advanced architectures focusing directly on early-stage ONFH, rather than just general staging, have also been reported. For example, Yang et al. [14] proposed a two-stage three-dimensional approach (3D-ONFHNet) for automatic staging between Ficat–Arlet stages 0–II with early ONFH detection and reported 93.83% accuracy in 381 MRI volumes from four centers. However, stage I cases were included in a limited number in this study, and the high computational load and increased memory load of three-dimensional architectures created significant limitations in terms of clinical integration. Uemura et al. [15] focused on automating the Steinberg volumetric classification, one of the indicators of collapse risk. For this purpose, a classification based on necrotic areas and femoral head segmentation was developed using Dynamic U-Net. However, the fact that the study only included pre-collapse cases and was single-center raises questions about its widespread applicability in different protocols.

Segmentation and quantitative studies are also being conducted using MRI images. Ruckli et al. [17] facilitated the surgical decision-making process by segmenting healthy and necrotic bone sections with 2D-3D segmentation using the nnU-Net base. Gao et al. [16] aimed to make inferences with 3D ROI radiographic features using nnU-Net and to estimate collapse risk with LightGBM in their study and reported AUC = 0.851 in the external test. In these studies, although it is important as an infrastructure for medical decision support, the direct staging decision differs methodologically compared to other models.

In addition, Vezirhüyük et al. [18] examined the consistency of different staging systems comparatively; however, the limited sample size and the lack of correlation with clinical outcomes reduce the power of the results obtained. While the literature generally reports high accuracy/AUC values on specific datasets, four common gaps stand out in terms of clinical application: (i) generalizability problems due to the small number of early-stage (especially stage I) image slices, (ii) the dominance of single-center/retrospective designs and the limitations of prospective validation, (iii) clinical integration difficulties due to computational-memory costs in 3D architectures, and (iv) the risk of inter-observer variability appearing as “tag noise” in the model in tasks open to interpretation, such as staging. Therefore, approaches that target Ficat staging and its binary distinction of early (I–II)/late (III–IV), evaluate different MRI planes separately, and aim to improve generalizability in a way that is compatible with clinical decision-making are particularly meaningful for real-world decision support.

## 2. Materials and Methods

**Figure 1:**
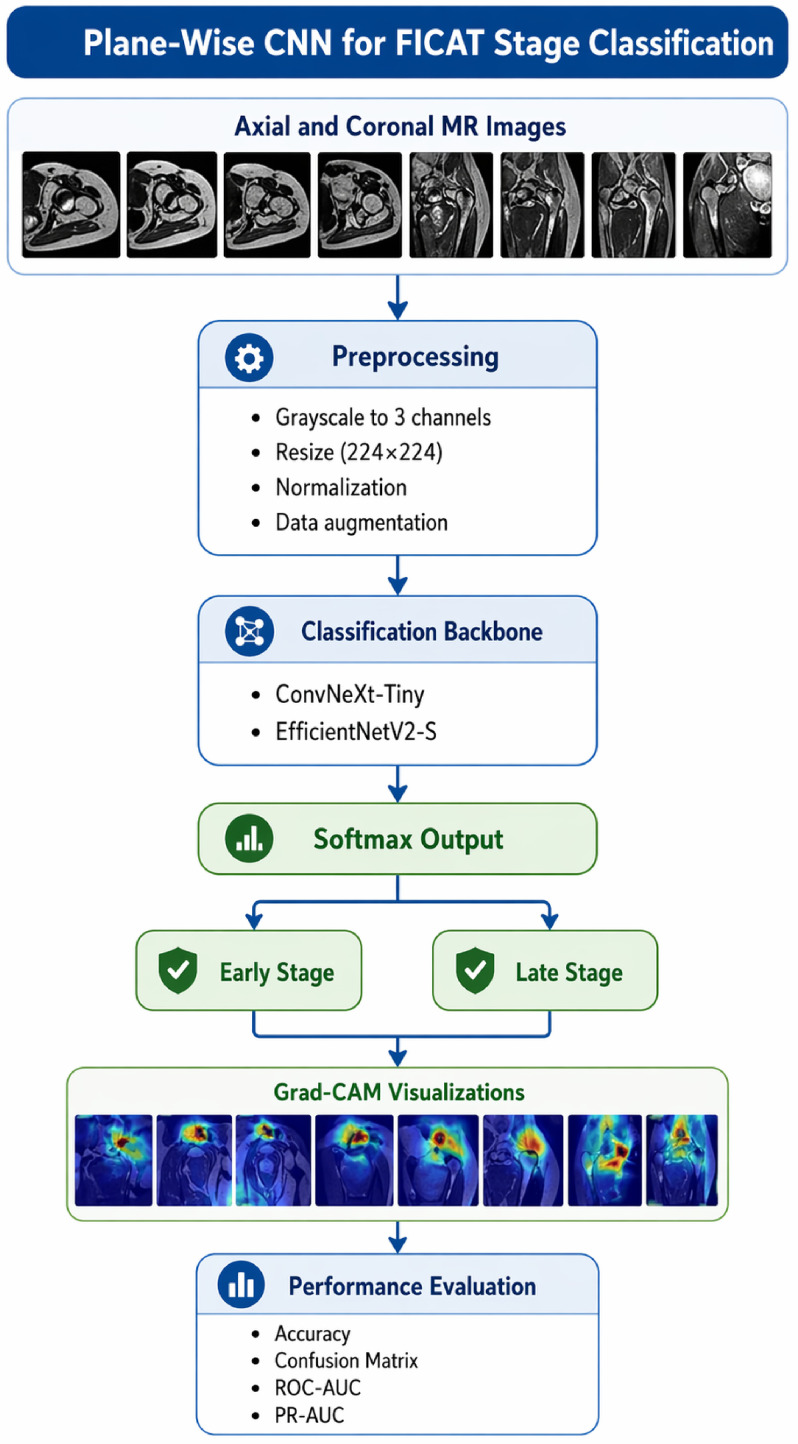
General workflow of the proposed deep learning-based ONFH staging model.

This study utilizes a plane-wise convolutional neural network approach for the Ficat stage classification problem. The aim of the proposed method is to classify ONFH cases into early- and late-stage groups by evaluating axial and coronal MR images separately. No feature-level fusion or patient-level multi-view integration was performed because paired axial–coronal images from the same patients were not available. The same classification pipeline was applied independently to each imaging plane, and the resulting performances were reported separately for axial and coronal images. Before model training, input MR images were converted into a three-channel format compatible with the network architectures and resized to the required input resolution. Intensity normalization was then applied to standardize the pixel-value scale and improve training stability. During training, augmentation techniques were used to improve generalization and reduce overfitting. No explicit segmentation was performed within the deep learning pipeline. In addition, no separate ROI-based cropping step was applied unless such cropping was already present in the curated dataset. Separate models were trained and evaluated for axial and coronal images using the same preprocessing and training protocol. In the feature extraction phase, feature maps obtained from axial and coronal images are represented through deep network backbones. For this purpose, high-level distinguishing features were obtained separately from axial and coronal images using ConvNeXt Tiny and EfficientNetV2 S architectures [19]. Final classification is performed with a softmax-based output layer, and probability values are generated for each class. The class with the highest probability is reported as the model’s prediction. Figure 1 provides a general overview of the proposed method.

The method’s performance is evaluated using several performance metrics. Overall classification performance is reported using the accuracy metric, while a confusion matrix is used to examine error types on a class basis. The area under the ROC curve is calculated to measure the discrimination power independent of the threshold value. The area under the PR curve, summarizing the relationship between precision and sensitivity, is also used as an additional evaluation metric, considering the possibility of class imbalance. This evaluation framework aims to reveal not only the average success level of the method but also its inter-class discrimination capacity and its resilience to potential data imbalances.

### 2.1. Preprocessing

The raw MR images were first converted into a three-channel format and resized to 224 × 224 pixels to ensure compatibility with the deep learning architectures used in this study. Intensity normalization was then applied to reduce variations in signal range across images and to provide a more standardized input for training. No explicit segmentation of the femoral head or necrotic area was performed before classification. Similarly, no additional ROI-based cropping centered on the femoral head was applied during preprocessing, unless such cropping was already present in the curated dataset. Slice selection was determined during dataset preparation, where the slice showing the clearest visualization of the femoral head and the most evident subchondral involvement was chosen for analysis; when needed, adjacent slices were also reviewed to support the staging decision [20].

### 2.2. Model Architecture

This study aims to automatically detect whether MR images belong to two distinct phases of clinical significance. The problem is defined as a binary classification problem addressed under a supervised learning approach. The dataset is defined as a sample set consisting of a limited number of image label pairs:

(1) $$D=\{\left(x_{i},\;y_{i}\right)|i=1,2,\ldots ,N\}$$

Here, $x_{i}$ represents a high-dimensional medical image, and $y_{i}\in \{0,1\}$ represents the corresponding clinical stage label. The main goal is to learn a generalizable function that associates images in the data field with labels in the output field. Medical images can have wide density distributions due to different devices, imaging parameters, and patient characteristics. This can lead to numerical instability in the learning process and overfitting of the model on certain samples. To reduce this effect, all images have been statistically normalized.

The normalized representation for each image sample is expressed as follows [20]:

(2) $${\tilde{x}}_{i}=\frac{x_{i}-\mu }{\sigma }$$

Here, *μ* and *σ* represent the mean and standard deviation values calculated over the entire dataset. This process ensures that different samples are represented in a common statistical space, contributing to a more stable optimization process.

### 2.3. Representation of Hierarchical Characteristics

Medical images contain multi-layered information, ranging from low-level structural features (edges, density transitions) to high-level semantic structures (anatomical and pathological patterns). In this study, a nonlinear, hierarchical transformation function was used to model this multi-level information [21].

This transformation is expressed as follows:

(3) $$h_{i}=\Phi \left({\tilde{x}}_{i};\Theta \right)$$

Here, Φ(⋅) represents a multi-layered transformation that extracts distinctive representations from the image, and Θ represents the set of parameters optimized during the learning process. Based on this structure, the model can interpret local patterns within a global context and learn representations that strengthen the distinction between classes. The learned high-level representations are projected onto the decision space for classification purposes. This projection is expressed by a linear transformation [22]:

(4) $$z_{i}=Wh_{i}+b$$

The values obtained at this stage represent the discrimination scores for each class. The magnitude of the scores indicates the proximity to the relevant class but does not yet contain a probabilistic interpretation. To make the decision scores clinically interpretable, they need to be converted into probability distributions. For this purpose, the scores are normalized to obtain class-conditional probabilities [23]:

(5) $$P(c|x_{i})=\frac{\exp\left(z_{i,c}\right)}{\sum_{k=1}^{2}exp\left(z_{i,k}\right)}$$

This definition guarantees that the sum of the probabilities of each image belonging to either class is equal to one. This makes uncertainty analysis and threshold-based decision mechanisms possible. One of the fundamental problems frequently encountered in medical datasets is the imbalance in the number of samples between classes. This can lead to biased results favoring the dominant class during the learning process. To mitigate this effect, a weighted risk function has been used:

(6) $$L=-\frac{1}{N}\sum_{i=1}^{N}\sum_{C=1}^{2}\omega_{C}∥\left(y_{i}=c\right)\log P\left(y_{i}=c\left|x_{i}\right.\right)$$

Here, $\omega_{c}$ is the coefficient that balances the contribution of the relevant class to the learning process. This approach aims to ensure that rare classes are adequately represented by the model. Model parameters have been updated using an adaptive and moment-based optimization strategy. The general parameter update rule is expressed as follows:

(7) $$\Theta_{t+1}=\Theta_{t}-\eta_{t}\left(\frac{{\hat{m}}_{t}}{\sqrt{{\hat{v}}_{t}+\varepsilon }}+\lambda \Theta_{t}\right)$$

This formulation is effective in terms of both rapid convergence and preventing overlearning. The regularization term increases the generalizability of the model by preventing excessive parameter growth. Keeping the learning rate constant can lead to performance loss at different stages of the training process. Therefore, a two-stage scheduling strategy has been adopted. In the first stage, the learning rate is gradually increased:

(8) $$\eta_{t}=\eta_{max}.\frac{t}{T_{\omega }},\;\;\;\;t<T_{\omega }$$

In the second stage, the learning rate was smoothly reduced using the cosine function:

(9) $$\eta_{t}=\eta_{min}.\frac{1}{2}\left(\eta_{max}-\eta_{min}\right)\left(1+cos\left(\pi \frac{t-T_{\omega }}{{T-T}_{\omega }}\right)\right)$$

This approach allows the model to make sufficient explorations in the early stages and reach a stable convergence in the late stages. Based on the probability distribution generated by the model, the final class label is determined using the maximum likelihood principle [24]:

(10) $${\hat{Y}}_{i}=\arg\;\underset{c}{\max}P\left(Y_{i}=c\left|x_{i}\right.\right)$$

This decision rule is a practical application of the Bayesian optimal classification approach. Due to the clinical significance of the positive class, the decision threshold was optimized using ROC analysis [25]:

(11) $$\tau^{*}=\arg\;\underset{\tau }{\max}\left(TPR\left(\tau \right)-FPR\left(\tau \right)\right)$$

Classification is done using this threshold:

(12) $${\hat{Y}}_{i}=\left\{\begin{array}{cc} 1, & \;\;P\left(Y_{i}=1\left|x_{i}\right.\right)\geq \tau^{*}\\ 0, & \;\;\;otherwise \end{array}\right.$$

This approach aims to improve clinical reliability by keeping the false negative rate under control. Interpretable model outputs are critical in medical practice. Therefore, the model’s decision-making process has been visualized using class-sensitive activation maps:

(13) $$H^{c}=ReLU\left(\sum_{k}\alpha_{k}^{c}F^{k}\right)$$

These maps reveal which spatial regions of the image the model focuses on when making decisions.

## 3. Experimental Studies

This section presents the experimental evaluation of the proposed deep learning-based method for ONFH staging. The model was evaluated on the test set by distinguishing early-stage disease, corresponding to Stages I–II, from late-stage disease, corresponding to Stages III–IV, using axial and coronal MRI images separately. To avoid potential data leakage, only one representative MR slice from each patient was included in the dataset. Therefore, although the train–test split was performed on an image basis, it was effectively equivalent to a patient-level split because each image belonged to a different patient. Thus, no images from the same patient appeared in both the training and test sets. All images were first shuffled and then randomly split into 80% training and 20% testing sets. This approach was intended to help the model learn from a wide range of images and to evaluate its performance effectively. ConvNeXt Tiny was used as the main classification model in the experimental studies. All images were resized to 224 × 224 pixels and trained with mini-batches of 32 samples over 40 epochs. During training, the learning rate was set to 0.0002, and the weight decay coefficient was set to 0.0005. The randomness constant was chosen as 42 for reproducibility purposes. To prevent overfitting, early stopping with a patience of 8 epochs was applied as the warm-up phase, and the learning rate was planned to be reduced to a minimum value of 0.000001. In the preprocessing stage, the images were converted to three-channel grayscale, and data augmentation operations such as rotation up to 10 degrees, shifting by 8%, scaling in the range of 0.95–1.10, and horizontal flipping with a 50% probability were applied to the training data. In addition, mean values of 0.5 and standard deviation values of 0.25 were used for normalization, and the late stage was considered as the positive class. These hyperparameters were selected based on preliminary experiments, computational feasibility, and commonly used settings in transfer learning-based medical image classification studies. The learning rate was chosen to provide stable convergence, while the batch size was selected according to GPU memory limitations and training stability. Early stopping was used to reduce overfitting.

### 3.1. Dataset Construction and Patient Selection

The dataset used in this study consisted of hip MRI examinations obtained from patients diagnosed with ONFH and treated at a tertiary healthcare center in Elazığ Province. The study was conducted after approval had been obtained from the relevant ethics committee for research involving human subjects. Initially, hip MRI examinations from 2600 patients were evaluated. The inclusion criteria were imaging performed due to hip pain, a clinically and radiologically confirmed diagnosis of ONFH, MRI examinations of sufficient image quality, and complete Ficat–Arlet staging information. Patients were excluded if they had severe motion artifacts, incomplete imaging sequences, insufficient visualization of the femoral head, missing clinical or staging data, a history of previous hip surgery, or concomitant hip pathologies such as tumor or infection. After these criteria were applied, 1843 patients were included in the final dataset, whereas 757 patients were excluded. A patient selection flowchart was added to make the dataset construction process clear, traceable, and reproducible, thereby improving reporting transparency. The patient cohort included individuals between 48 and 69 years of age. Dataset construction involved patient identification, eligibility assessment, image-quality control, Ficat–Arlet staging, representative slice selection, and final allocation to the training and test sets. Disease staging was performed according to the Ficat–Arlet classification system, based on both clinical and radiological findings. Direct MRI findings were also considered during the staging process. Stages I and II were categorized as early-stage ONFH, whereas Stages III and IV were categorized as late-stage ONFH. Accordingly, a deep learning-based binary classification approach was employed to distinguish early-stage from late-stage disease. The distribution of cases across stages showed a noticeable imbalance. In the early-stage group, imaging findings were generally subtle, whereas femoral head collapse and joint degeneration were more evident in the late-stage group. In terms of imaging plane, the early-stage group included 1044 axial images and 1172 coronal images, resulting in a total of 2216 images. The late-stage group included 469 axial images and 671 coronal images, resulting in a total of 1140 images. Overall, the dataset included 1513 axial images and 1843 coronal images, indicating that coronal images were more abundant than axial images and that early-stage samples outnumbered late-stage samples. MRI was performed using a 1.5 Tesla scanner (Siemens Healthcare, Erlangen, Germany) according to a standardized imaging protocol. The protocol included T1-weighted, T2-weighted, and fat-suppressed sequences acquired in the axial, coronal, and sagittal planes. Slice thickness ranged from 3 to 4 mm, with an interslice gap ranging from 0.5 to 1 mm. Representative slice selection was performed according to a predefined protocol. For each patient, multiple consecutive MRI slices including the femoral head were systematically reviewed. The slice selected for analysis was the one that provided the clearest visualization of the femoral head and showed the most prominent subchondral involvement. After this selection process, only one representative MRI image was retained for each patient within each plane-based dataset. Therefore, the same patient was not represented by multiple slices within the same axial or coronal experiment. When necessary, adjacent slices were also examined to improve the accuracy of the assessment. Ficat–Arlet staging was performed independently and in a blinded manner by two orthopedic and traumatology specialists, without access to patients’ clinical information. Each observer evaluated the MRI examinations separately and assigned stages independently. In cases of disagreement, the images were jointly re-evaluated until consensus was reached. This approach was adopted to minimize observer-related bias during the assessment process.

### 3.2. Axial Plane Results

**Figure 2:**
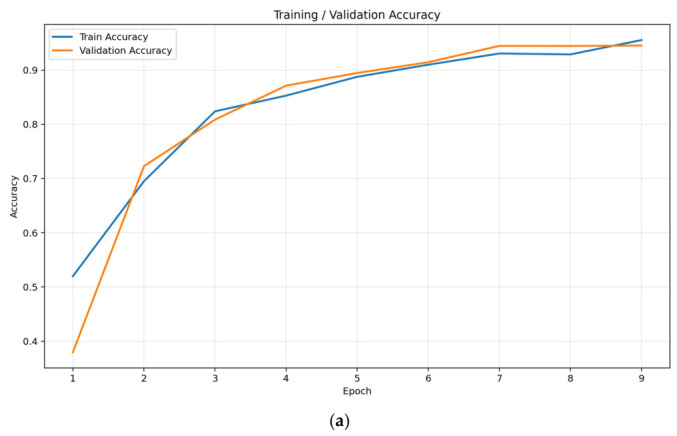

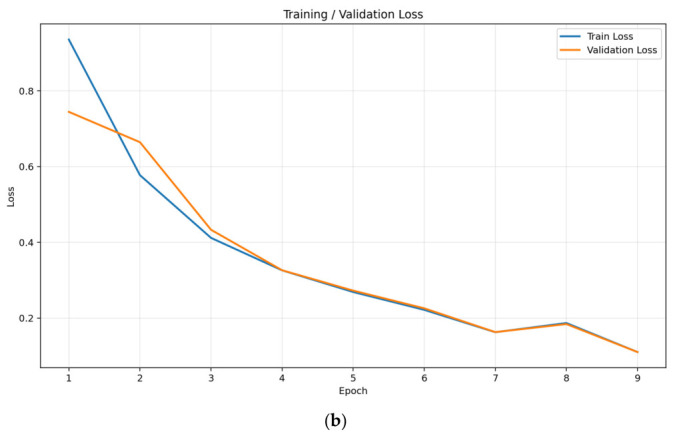
Training and loss curves for axial view images (**a**) Accuracy, (**b**) Loss.

The test results obtained in the axial plane are presented in Table 2. Figure 2 also illustrates the training and validation accuracy and loss curves across epochs. The developed model demonstrated high classification performance in distinguishing between early and late stages. Accuracy of 94.51%, sensitivity of 96.80%, and specificity of 93.49% were achieved on the test set. The model’s F1 score was calculated as 0.9162, and the area under the ROC curve (AUC) was 0.981.

**Figure 3:**
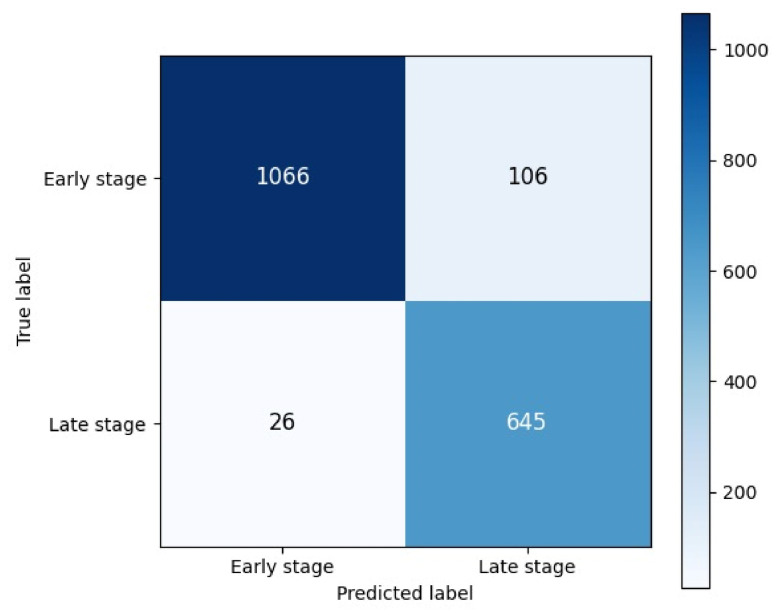
Confusion matrix obtained for early- and late-stage ONFH classification of Axial plane.

Examining the confusion matrix, 1066 examples belonging to the early stage were correctly classified, while 106 examples were misclassified; 645 examples belonging to the late stage were correctly classified, while 26 examples were misclassified. These results show that the model can distinguish late-stage ONFH cases with high accuracy. In this study, the positive class was defined as late-stage ONFH cases. A visual summary of the confusion matrix and classification performance is provided in Figure 3, highlighting the distribution of correct and incorrect predictions for both early and late-stage cases.

This confusion matrix shows the model’s performance in distinguishing between early-stage and late-stage ONFH cases. Of the early-stage cases, 1066 were correctly classified and 106 were incorrectly classified. In late-stage cases, there were 645 correct classifications and only 26 incorrect classifications. This distribution indicates that the model can identify late-stage ONFH cases with high accuracy, while making a limited number of errors in the early stage. The low number of false negatives shows that clinically critical advanced-stage cases are largely correctly identified. Figure 3 presents the confusion matrix for early and late-stage ONFH classification.

**Figure 4:**
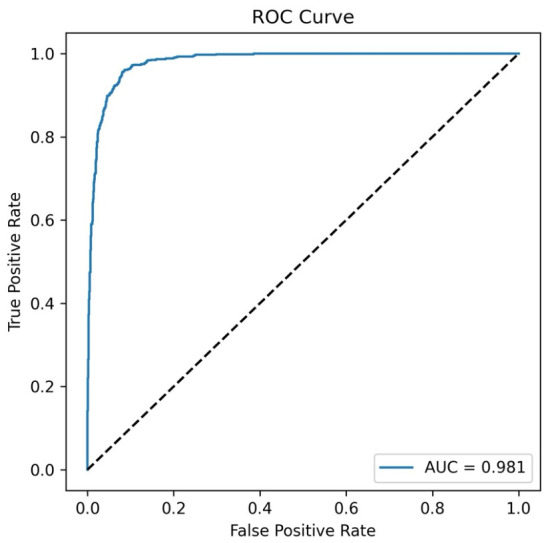
ROC curve of the model for early- and late-stage ONFH classification based on axial plane MRI images (AUC = 0.981).

Figure 4 presents the ROC curve and classification performance obtained in the axial plane experiment. This ROC curve demonstrates the model’s discriminatory power in distinguishing between early and late-stage ONFHs. The curve’s proximity to the upper left corner and the AUC value of 0.981 indicate that the model provides high sensitivity and specificity. This result shows that the model is significantly superior to random classification.

### 3.3. Coronal Plane Results

In tests performed in the coronal plane, as shown in Table 3, the model achieved accuracy of 92.84%, sensitivity of 96.13%, and specificity of 90.96%. The F1 score was calculated as 0.9072, and the AUC value as 0.988. According to the confusion matrix results, 976 examples were correctly classified and 68 examples were incorrect in the early stage class; and 454 examples were correctly classified and 15 examples were incorrect in the late stage class. Although the results obtained in the coronal plane are relatively lower compared to the axial plane, they demonstrate clinically acceptable and strong performance.

**Figure 5:**
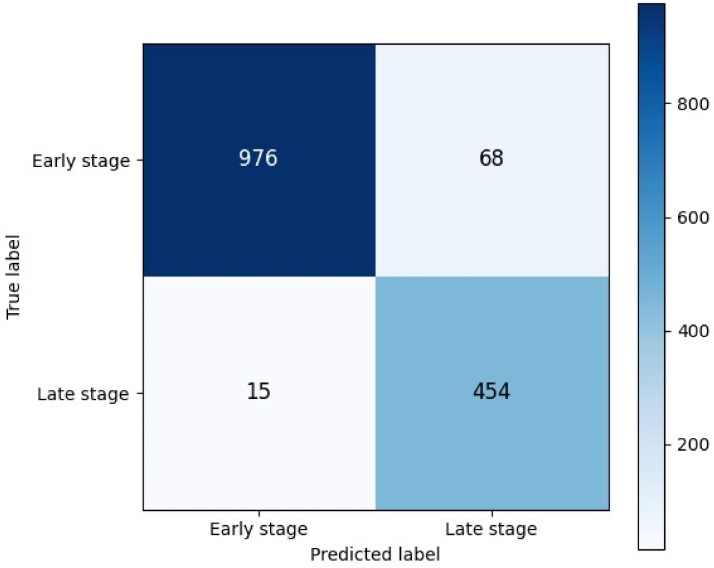
Confusion matrix of early- and late-stage ONFH classification for coronal plane.

Figure 5 presents the confusion matrix of early- and late-stage ONFH classification. As seen in Figure 5, 976 early-stage cases were correctly classified, and 68 were incorrectly classified. In late-stage cases, there were 454 correct and 15 incorrect classifications. These findings indicate that the coronal plane model achieved reliable discrimination between early- and late-stage ONFH cases, with a low number of misclassified late-stage samples. The low error rate, especially in the late stage, suggests that the model more consistently captures advanced pathological changes.

**Figure 6:**
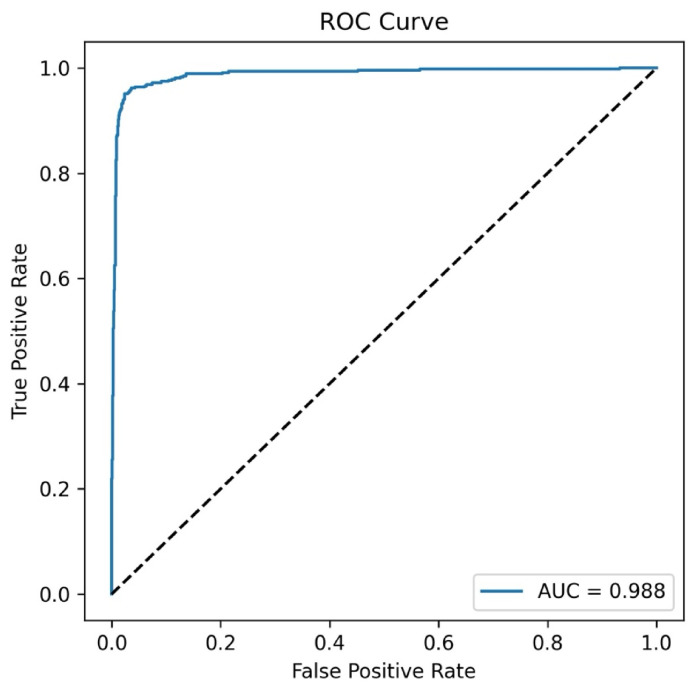
ROC curve of the model for early- and late-stage ONFH classification based on coronal plane MRI images (AUC = 0.988).

Figure 6 shows the obtained ROC curve. As shown in Figure 6, the ROC curve illustrates the model’s discrimination capacity between early and late-stage ONFH cases. The AUC value of 0.988 indicates strong discriminatory capacity and supports the potential of the model as a reliable clinical decision support tool. The AUC value of 0.988 indicates strong discriminatory capacity and suggests that the model maintained stable performance across the evaluated data distribution. This finding supports the idea that the model can be a reliable decision support tool in clinical applications.

### 3.4. Comparison of Axial and Coronal Planes

Axial and coronal plane results are presented in Table 4. The axial plane model achieved higher accuracy, sensitivity, specificity, and F1-score than the coronal plane model, whereas the coronal plane model showed a slightly higher AUC value. This indicates that axial images provided stronger threshold-dependent classification performance, while coronal images demonstrated slightly higher threshold-independent discrimination capacity. Therefore, both planes provided clinically meaningful but plane-dependent classification performance.

In the experimental studies, we also used 10-fold cross-validation test for both axial and coronal images to provide a more reliable evaluation of model performance. In this approach, the dataset was divided into ten subsets, and in each iteration, one subset was used for validation/testing while the remaining subsets were used for training. This procedure allowed the model to be assessed under different data splits and helped examine its stability and generalizability for both axial and coronal views. Table 5 shows the obtained evaluation metrics for each fold.

Table 5 presents the 10-fold cross-validation results for early- and late-stage ONFH classification in the coronal plane. As seen in Table 5, the model showed a stable and consistent performance across the different folds, with accuracy values ranging from 84.78% to 90.81%. The mean accuracy was 87.57 ± 2.11%, while the mean sensitivity and specificity were 85.54 ± 4.83% and 88.74 ± 3.28%, respectively. The average F1-score was 0.8336 ± 0.0283, indicating a balanced classification performance between the early and late stage groups.

Table 6 presents the 10-fold cross-validation results for early- and late-stage ONFH classification in the axial plane. As shown in Table 6, the model demonstrated stable and consistent performance across the different folds, with accuracy values ranging from 87.50% to 94.04%. The mean accuracy was 90.62 ± 2.16%, while the mean sensitivity and specificity were 85.08 ± 6.51% and 93.10 ± 2.02%, respectively. The average F1-score was 0.8482 ± 0.0377, indicating a balanced and reliable classification performance between the early and late stage groups in the axial plane.

The difference between the hold-out test results and the 10-fold cross-validation results may be attributed to the variability of data partitioning in a limited and imbalanced medical dataset. The hold-out test represents the performance obtained from a single fixed split, whereas 10-fold cross-validation evaluates the model across multiple different train–test partitions. Therefore, the slightly lower mean performance in cross-validation provides a more conservative estimate of generalization performance. Importantly, the cross-validation results still demonstrate stable and clinically acceptable performance across folds, supporting the robustness of the proposed model.

### 3.5. Explainability of the Model

**Figure 7:**
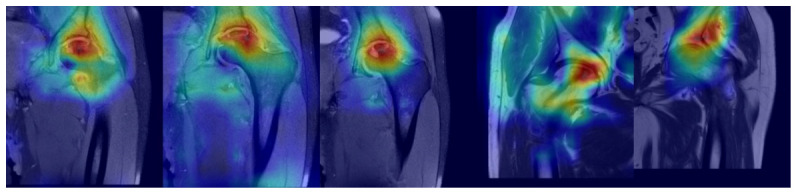
Grad-CAM heat map visualizations on MR images of early-stage ONFH cases.

In this study, the performance of the developed deep learning model was evaluated using confusion matrices, ROC curves, and Grad-CAM-based visual explainability analyses. Confusion matrices detail the model’s success in distinguishing between early-stage and late-stage ONFH cases. The high number of correctly classified examples indicates consistent performance in both stage groups. The relatively low number of incorrectly classified examples supports the model’s high inter-stage discrimination power. ROC curves and their corresponding AUC values quantitatively reveal the model’s discrimination capacity. The fact that the obtained ROC curves are close to the ideal upper-left corner and the AUC values are above 0.98 shows that the developed model provides high sensitivity and specificity in distinguishing between early and late-stage ONFH. These outcomes show that the model significantly outperforms random classification and may have potential use as a clinical decision support system. To help clarify the decision process used by the model, Grad-CAM was used to create visualizations. The heat maps show that the model primarily focuses on the subchondral and weight-bearing areas of the femoral head during classification. This shows that these areas are the most relevant to ONFH pathophysiology, as necrosis and changes in these areas are commonly the first to occur in ONFH. The model also seems to focus on similar anatomical areas throughout different slices of the image, indicating that the model’s decisions are not random but are based on identifiable image features. Figure 7 presents Grad-CAM-based attention maps for MR images from cases of early-stage ONFH.

**Figure 8:**
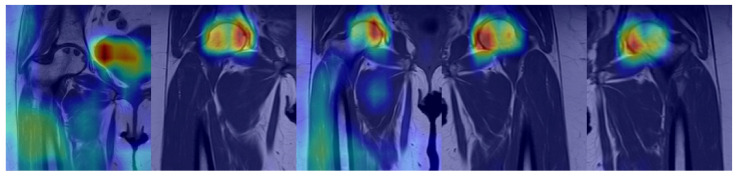
Grad-CAM based attention maps on MR images of late-stage ONFH cases.

Figure 8 presents Grad-CAM-based attention maps for MR images from cases of late-stage ONFH. As can be noted from these Grad-CAM images, the model has focused its attention on the subchondral areas as well as the areas that bear the weight of the femoral head while making its decision for classification. The areas highlighted in red and yellow represent the areas that play a major role in the decision-making process by the model. The overlap of these areas with the areas that experience ischemic changes and necrosis in ONFH cases indicates that the model has learned some meaningful features from the images.

The emphasis on similar anatomical regions in different sections of these images demonstrates that the model’s decisions are not random and are shaped by consistent visual patterns. The concentration of activity in the anterosuperior section of the femoral head suggests that the model accurately identifies areas with a high risk of collapse.

## 4. Discussion

This study proposed a deep learning-based approach for the classification of early- and late-stage ONFH using axial and coronal MRI images. A transfer learning approach was adopted, leveraging the model’s original visual feature learning capacity and enabling its adaptation to magnetic resonance imaging. The architectural structure generates hierarchical feature representations from images through multi-stage convolution blocks; it models edge and density transitions in superficial layers and more complex pathological patterns in the subchondral area and weight-bearing regions of the femoral head in deep layers. The resulting high-level feature maps are summarized using global pooling. Early and late-stage classification is performed via a fully connected decision layer. To prevent bias in the learning process due to class disproportion, a weighted loss function was used. Furthermore, the decision threshold was optimized based on ROC analysis, depending on the clinical significance of the positive class, with the specific goal of reducing the false negative rate. This methodological approach provides a holistic classification method that combines deep representational learning with clinical decision reliability. In this study, both convolutional neural network-based and transformer-based deep learning architectures were compared using the proposed method. All models were classified into training and validation subsets while maintaining class distribution based on a similar data partitioning strategy, and trained within the same preprocessing stages and training protocol to create an unbiased comparison environment. Before training, images were adapted from grayscale to three-channel format, rescaled, and normalized. During the training process, data augmentation approaches such as random transformation, random linear transformation, and horizontal flipping were implemented to increase the generalizability potential of the model. EfficientNetV2-S, DenseNet-121, ResNet-18, ConvNeXt-Tiny, and Swin-Tiny architectures were preferred for model comparisons. EfficientNetV2-S was analyzed as a next-generation CNN architecture with high parameter efficiency; DenseNet-121 and ResNet-18 were analyzed as traditional CNN architectures generally cited in scientific literature. ConvNeXt-Tiny represents a current architecture encompassing modern CNN design principles, while Swin-Tiny represents the transformer class with its window-based cascaded transformer architecture. Accordingly, traditional CNN, current CNN, and transformer-based approaches were compared on the same dataset.

All models were trained for 40 epochs with a batch size of 32. The AdamW algorithm was used for optimization, and the initial learning rate was set at 2 × 10^−4^. A cosine annealing strategy with warm-up was preferred in learning rate planning. Class-weighted Cross-Entropy was used in the loss function to reduce the effect of class imbalance. Training was terminated when no improvement in validation performance was observed with the early stopping mechanism. Table 7 shows the results obtained for the axial plane. Examining the axial plane results, it was observed that the Swin-Tiny model showed the highest performance with accuracy of 91.42%, specificity of 90.60%, and F1 score of 89.79%. In terms of sensitivity, the ConvNeXt-Tiny model achieved the highest performance with a value of 90.83%. These findings demonstrate that transformer-based architectures can learn more discriminatory feature representations in the axial plane, whereas modern CNN architectures exhibit strong performance, particularly in determining the positive class, by providing high sensitivity.

Table 8 also shows the results obtained for the coronal plane. When the coronal plane results were examined, it was observed that the DenseNet-121 model provided the most balanced and highest performance with accuracy of 89.13, sensitivity of 88.58, specificity of 88.12, and F1 score of 88.34. The fact that the superior performance of the Swin-Tiny model in the axial plane did not continue at the same level in the coronal plane suggests that plane-dependent anatomical variations and morphological features may affect the representational power of model architectures in different ways.

The proposed ConvNeXt Tiny-based pipeline achieved higher performance than the compared baseline architectures in both axial and coronal plane experiments. In the axial plane, accuracy was 94.51%, sensitivity 96.80%, specificity 93.49%, and F1-score 91.62%. These values exceeded those of the Swin-Tiny ConvNeXt-Tiny DenseNet-121 and ResNet-18 models. The high sensitivity of 96.80% suggests that our model can provide higher clinical reliability in accurately identifying both early and late-stage cases. In the coronal plane, accuracy was calculated as 92.84%, sensitivity as 96.13%, specificity as 90.96%, and F1-score as 90.72%. These results indicate a higher overall classification performance compared to other CNN and transformer-based approaches, particularly DenseNet-121. The findings demonstrate that the proposed method possesses a more balanced and robust representation learning capacity across different anatomical planes.

Advantages of the proposed method:

1. The proposed method produced high discrimination performance for early-stage and late-stage ONFH.
2. Grad-CAM images have shown that the model focuses on clinically significant regions.

Additionally, the limitations of the proposed method are:

1. The study was conducted with single-center and retrospective data. The model has not been subjected to external validation with data from different centers.

## 5. Conclusions

This study presents a plane-wise deep learning approach supported by an explainability layer, targeting the differentiation of early- and late-stage femoral head osteonecrosis from MR images. Experimental findings demonstrated that the proposed plane-wise approach achieved high classification performance in separate evaluations of axial and coronal MR images. High AUC values obtained through ROC analysis supported the model’s strong threshold-independent discrimination power. Grad-CAM-based visualizations revealed that the decision-making process was consistent with clinically significant regions of the femoral head. In this respect, the method can be considered a decision support approach that can reduce observer-dependent variability in staging. In future studies, external validation of the method with data from different centers and different imaging protocols is considered a priority. Integration into clinical workflows and real-time use scenarios should be tested with prospective studies. Multi-expert consensus labels and learning strategies modeling label ambiguity can be evaluated to reduce potential noise in stage labels. In addition, future studies may investigate true patient-level multi-plane fusion strategies using paired axial–coronal images from the same patients. In terms of explainability, quantitative explainability measures and clinical validation protocols should be added, going beyond simply presenting heat maps.

The proposed method may be most applicable as a decision support tool in settings where axial or coronal MR images are available and a binary early-versus-late ONFH distinction is clinically required. However, the method may fail or show reduced reliability in images obtained with substantially different MRI protocols, severe motion artifacts, poor femoral head visualization, postoperative morphological changes, uncommon anatomical variations, or borderline Stage II–III cases. Therefore, external validation and prospective clinical testing are necessary before routine clinical use.

## Figures and Tables

**Table 1:** Comparison of deep learning-based studies for the diagnosis of osteonecrosis of the femoral head (ONFH).

Author (Year)	Model/Method Used	Imaging Modality	Performance (%)	Disadvantages
Yang et al. (2025) [14]	3D-ONFHNet	MRI	93.83%	Despite high accuracy, the limited number of Stage I cases restricts the generalizability of early diagnosis. Furthermore, the 3D architecture makes clinical integration difficult due to high computational and memory requirements.
Shen et al. (2024) [12]	DenseNet-based CNN	MRI	87.8%	The retrospective design of the study and the lack of data in mild stages limit the reliability of the model in early ONFH diagnosis.
Gao et al. (2024) [16]	nnU-Net + Radiomic + LightGBM	MRI	76.5%	Image heterogeneity in multicenter data led to a decrease in model performance, especially in moderate-risk cases.
Ruckli et al. (2023) [17]	nnU-Net (2D–3D segmentation)	MRI	75%	The small, single-center dataset reduces the generalizability of the results; also, the study focuses on segmentation rather than diagnosis.
Klontzas et al. (2024) [13]	CNN Ensemble	MRI	85.5%	A decrease in external testing performance was observed due to the limited sample size, which restricted the clinical generalization of the model.
Vezirhüyük et al. (2025) [18]	Comparison of classification systems	MRI	65%	The study is limited to pre-collapse cases and is single-center, making its clinical applicability in advanced stages uncertain.
Uemura et al. (2025) [15]	Dynamic U-Net + Steinberg	MRI	93.7%	Despite high accuracy, the limited number of Stage I cases restricts the generalizability of early diagnosis. Furthermore, the 3D architecture makes clinical integration difficult due to high computational and memory requirements.

**Table 2:** Performance metrics for early and late-stage ONFH classification in the axial plane.

Accuracy (%)	Sensitivity (%)	Specificity (%)	F1-Score	AUC
94.51	96.80	93.49	0.9162	0.981

**Table 3:** Performance metrics for early- and late-stage ONFH classification in the coronal plane.

Accuracy (%)	Sensitivity (%)	Specificity (%)	F1-Score	AUC
92.84	96.13	90.96	0.9072	0.988

**Table 4:** Performance comparison of ONFH staging in axial and coronal planes.

Plane	Accuracy (%)	Sensitivity (%)	Specificity (%)	F1-Score	AUC
Axial	94.51	96.80	93.49	0.9162	0.981
Coronal	92.84	96.13	90.96	0.9072	0.988

**Table 5:** Performance metrics for early- and late-stage ONFH classification in the coronal plane for 10-fold cross validation test.

Fold	Accuracy (%)	Sensitivity (%)	Specificity (%)	F1-Score
1	90.81	83.58	94.92	0.8682
2	85.41	86.57	84.75	0.8112
3	85.95	86.76	85.47	0.8194
4	85.33	79.10	88.89	0.7970
5	89.13	85.07	91.45	0.8507
6	87.50	92.54	84.62	0.8435
7	88.04	86.57	88.89	0.8406
8	89.67	92.54	88.03	0.8671
9	89.13	85.07	91.45	0.8507
10	84.78	77.61	88.89	0.7879
Mean ± Std	87.57 ± 2.11	85.54 ± 4.83	88.74 ± 3.28	0.8336 ± 0.0283

**Table 6:** Performance metrics for early- and late-stage ONFH classification in the axial plane for 10-fold cross validation test.

Fold	Accuracy (%)	Sensitivity (%)	Specificity (%)	F1-Score
1	87.50	76.60	92.38	0.7912
2	88.82	74.47	95.24	0.8046
3	93.42	91.49	94.29	0.8958
4	94.04	89.13	96.19	0.9011
5	89.40	82.98	92.31	0.8298
6	89.40	78.72	94.23	0.8222
7	90.73	85.11	93.27	0.8511
8	88.74	89.36	88.46	0.8317
9	93.38	95.74	92.31	0.9000
10	90.73	87.23	92.31	0.8542
Mean ± Std	90.62 ± 2.16	85.08 ± 6.51	93.10 ± 2.02	0.8482 ± 0.0377

**Table 7:** Performance metrics for early and late-stage ONFH classification in the axial plane.

Model	Accuracy	Sensitivity	Specificity	F1-Score
EfficientNetV2-S	0.8548	0.8278	0.8421	0.8341
DenseNet 121	0.8911	0.8772	0.8708	0.8739
ResNet18	0.8845	0.8753	0.8614	0.8677
ConvNet-Tiny	0.8977	0.9083	0.8736	0.8863
Swin-Tiny	0.9142	0.8910	0.9060	0.8979

**Table 8:** Performance metrics of deep learning models for early and late-stage ONFH classification in the coronal plane.

Model	Accuracy	Sensitivity	Specificity	F1-Score
EfficientNetV2-S	0.8587	0.8506	0.8464	0.8484
DenseNet 121	0.8913	0.8858	0.8812	0.8834
ResNet18	0.8832	0.8810	0.8713	0.8755
ConvNet-Tiny	0.8832	0.8710	0.8906	0.8775
Swin-Tiny	0.8832	0.8683	0.8773	0.8724

## Data Availability

The data presented in this study are available on request from the corresponding author.
